# Stretchable Tattoo-Like Heater with On-Site Temperature Feedback Control

**DOI:** 10.3390/mi9040170

**Published:** 2018-04-08

**Authors:** Andrew Stier, Eshan Halekote, Andrew Mark, Shutao Qiao, Shixuan Yang, Kenneth Diller, Nanshu Lu

**Affiliations:** 1 Department of Electrical and Computer Engineering, University of Texas at Austin, Austin, TX 78712, USA; andrewcstier@gmail.com (A.S.); ehalekote@gmail.com (E.H.); andrewemark@gmail.com (A.M.); 2 Center for Mechanics of Solids, Structures and Materials, Department of Aerospace Engineering and Engineering Mechanics, University of Texas at Austin, Austin, TX 78712, USA; shutao2011@gmail.com (S.Q.); rock002008@gmail.com (S.Y.); 3 Department of Biomedical Engineering, University of Texas at Austin, Austin, TX 78712, USA; kdiller@mail.utexas.edu; 4 Texas Materials Institute, the University of Texas at Austin, Austin, TX 78712, USA

**Keywords:** epidermal electronics, wearable heater, temperature sensor, feedback control

## Abstract

Wearable tissue heaters can play many important roles in the medical field. They may be used for heat therapy, perioperative warming and controlled transdermal drug delivery, among other applications. State-of-the-art heaters are too bulky, rigid, or difficult to control to be able to maintain long-term wearability and safety. Recently, there has been progress in the development of stretchable heaters that may be attached directly to the skin surface, but they often use expensive materials or processes and take significant time to fabricate. Moreover, they lack continuously active, on-site, unobstructive temperature feedback control, which is critical for accommodating the dynamic temperatures required for most medical applications. We have developed, fabricated and tested a cost-effective, large area, ultra-thin and ultra-soft tattoo-like heater that has autonomous proportional-integral-derivative (PID) temperature control. The device comprises a stretchable aluminum heater and a stretchable gold resistance temperature detector (RTD) on a soft medical tape as fabricated using the cost and time effective “cut-and-paste” method. It can be noninvasively laminated onto human skin and can follow skin deformation during flexure without imposing any constraint. We demonstrate the device’s ability to maintain a target temperature typical of medical uses over extended durations of time and to accurately adjust to a new set point in process. The cost of the device is low enough to justify disposable use.

## 1. Introduction

There exists a need for soft, stretchable electronic heating devices that can conform to human skin unobstructively and stay attached during long term use. Such devices can serve a variety of applications in the medical field. As some examples, heat is commonly used in physical therapy following exercise-induced delayed onset muscle soreness (DOMS) [1,2]. Heating injured joints can induce thermal expansion of the collagen tissue and thus reduce pain and stiffness [3,4,5]. When hypothermia occurs due to anesthesia [6], applying heat to the palms and soles of a patient with distended blood vessels can re-warm the body’s core temperature [7,8]. Applying heat over a skin surface can accelerate the transdermal diffusion of chemicals from a drug patch [9,10].

Conventional heaters used to treat muscle pain or joint injuries include electric heat packs [11] and heat wraps [12]. Heat packs do not have very controllable temperature and are heavy and bulky. Heat wraps are easier to control but are also heavy and their rigidity makes it difficult for them to be worn seamlessly [5]. These products’ inability to conform well to skin [13,14,15] make them less comfortable and also present a more severe problem—lack of uniform and consistent adhesion to the skin surface could lead to air gaps which cause hotspots [16]. These hotspots could burn the skin if the heater is operated near the safety threshold of 43 °C [17]. This can severely limit the range and thus the effectiveness of the conventional heaters.

One heating method that can safely heat the body at temperatures close to 43 °C and the current gold standard for preventing the hypothermia caused by anesthesia, is forced air warming. Forced air warming heats air and pumps it into blankets covering large portions of the patient. While effective at raising the core body temperature, forced air warming has some disadvantages including bulkiness, obstructiveness to surgeries and high cost [18,19].

Recently there has been an expansion of the development of stretchable electronics [20,21,22,23]. Methods that have been used to produce these type of electronics include embedding carbon nanotubes (CNTs) in elastomers [24,25], depositing silver (Ag) nanoparticles in polyeruthane [5,26], chemically bonding Ag flakes to CNTs [27,28], combining Ag nanoparticles with elastomeric fibers [29], electrospinning Ag nanofibers onto a flexible substrate [30], constructing CuZr nanotrough networks that function as stretchable electrodes [31], constructing stretchable gold (Au) electrodes from multi-layers of Au nanosheets [32] and patterning metal thin films into serpentine [33,34] or fractal shapes [35,36] to minimize their strain during stretching. This last method has enabled the creation of epidermal electronics—ultrathin, ultrasoft electronics, physiological sensors, and electrical and thermal stimulators—that can adhere and conform to skin surfaces and bend and stretch without breaking, detaching, or imposing any mechanical constraint to the skin [36,37,38].

With the development of stretchable electronics, stretchable patch heaters have emerged in recent years. Examples include joule heating devices fabricated from soft Ag nanowire composites [5,30,39], copper (Cu) nanowire based fabric [40], stretchable copper zirconium electrodes [31], or stretchable Au serpentines [6,11,29,32]. Using a stretchable and conformable heater could solve the major disadvantages of conventional solid heaters. However, the existing stretchable heaters involve expensive nanomaterials or time-consuming procedures to produce. Moreover, most of them have no method of acquiring temperature feedback from the heater as they are not equipped with any temperature sensors. As a result, most of the reported stretchable heaters do not use temperature feedback to autonomously maintain a set temperature for the heater. One of the exceptions is a wearable fabric heater described by Cheng et al. [40]. This heater is loose enough that it is able to use an unspecified temperature sensor to monitor and control the heater. Of the more tightly conformable heaters, one with temperature feedback only has the functionality of turning the heater off if it gets too hot—aside from that, the temperature feedback is not used to actively control the heat of the heater [9]. The best existing example of a tightly conforming stretchable heater with continuous feedback control is the metal nanofiber heater developed by Jang et al. [30]. This heater uses a thermistor placed on the outer edge of the heater to detect the heater’s temperature and uses an unspecified control algorithm to keep the heater at a specified temperature. This set-up relies on the assumption that the temperature at the outer edge of the heater is representative of the temperature of the heater overall but the heat profile across the heater is not actually uniform. Moreover, the thermistor appears to introduce disturbances in the uniformity of the heater.

In other stretchable heaters with temperature sensors, the temperature sensing element is the same as the heating element [9,15]. The biggest disadvantage of this setup is that the dual-purpose element is measuring its own temperature instead of the actual skin temperature. The closest example of a stretchable heater with separate, unobstructive temperature feedback is one developed for a prosthetic hand but not tested on human skin, where a multilayer heater and sensor array is laminated onto a prosthetic hand [41]. Without the use of effective temperature feedback, past stretchable heating devices have relied on the relationship between voltage and heat generated in order to maintain the heater at a desired temperature. However, heat transfer conditions vary from person to person and it is inaccurate to assume a consistent relationship between voltage and temperature if you wish to apply the same heater to multiple subjects. For example, changes in blood flow can cause changes in epidermal skin temperature [7].

Our group has developed a “cut-and-paste” method [42], in which stretchable patterns are cut out of ultrathin metal-polymer laminates and pasted to an adhesive substrate, allowing for cheaper, quicker and greener fabrication of tattoo-like sensors. This method also allows for easy integration of independent heaters and resistance temperature detectors (RTDs) on the same substrate. Using this method, we herein present an inexpensive, easy to fabricate and power-efficient programmable tattoo-like heating device which comprises a stretchable resistive heating element (RHE) of serpentine-shaped aluminum (Al) ribbons and a stretchable RTD of serpentine-shaped Au ribbons. The RTD is thin enough to not disturb the uniformity of the heat from the heating element. Included with this device is a customized proportional-integral-derivative (PID) control software which uses real time temperature feedback to control the heater and can maintain it at a target temperature over a large area of skin for extended periods of time.

## 2. Materials and Methods

**Figure 1:**
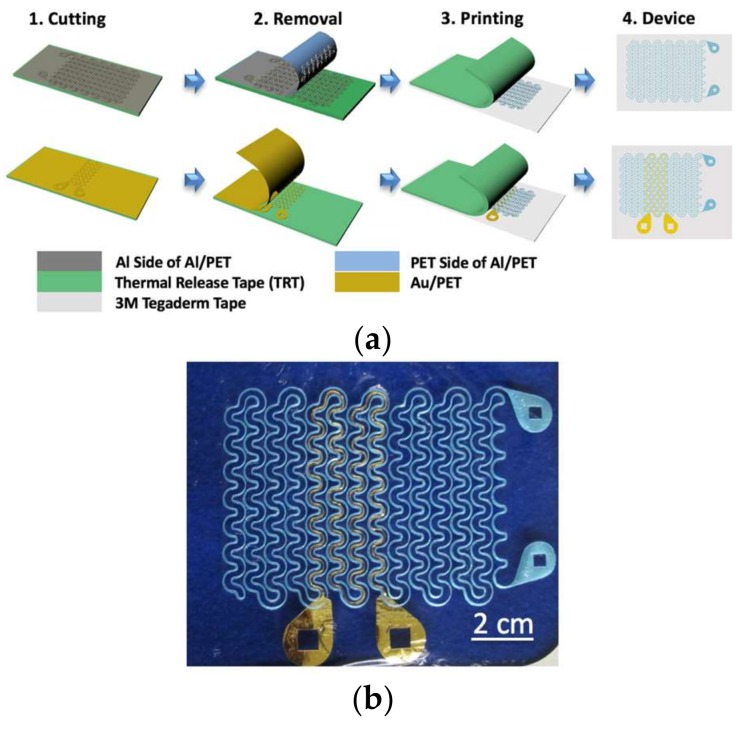
(**a**) Fabrication process used for heater and resistance temperature detector (RTD), shown for heater. Material is put on the thermal release tape (TRT) and cut with Silhouette cutter. TRT is heated, excess material is removed and remaining material is transferred to Tegaderm; (**b**) Complete device on tegaderm. Aluminum with blue polyimide backing forms the resistive heating element while Au/Cr 100/10 nm forms the resistance temperature detector.

The “cut-and-paste” manufacturing process of the stretchable tattoo-like heater is illustrated in Figure 1a, and a picture of the as-fabricated sample on 3M Tegaderm tape is offered in Figure 1b. As depicted in the first row of Figure 1a, the process began with placing a blanket 7 µm/13 µm Al/PET bilayer laminate (Neptco Inc., Pawtucket, RI, USA) smoothly on a thermal release tape (Semiconductor Equipment Corp., Moorpark, CA, USA) with Al facing up. A Silhouette mechanical cutter plotter was programmed to cut the designed seams on the bilayer within 3 min. Excessive Al/PET was removed once the thermal release tape (TRT) was heated and the remaining Al/PET ribbon was printed on a 3M Tegaderm tape with the Al side facing the Tegaderm and the bluish PET side facing outward. The 13-µm thick PET layer allows for increased mechanical integrity [43] and electrical insulation. The same process was repeated to cut and paste the stretchable RTD ribbon, which was made out of 100 nm/15 nm/13 µm Au/Cr/PET laminate, with the PET facing the Tegaderm and the Au facing outward, as illustrated by the second row of Figure 1a. This arrangement resulted in two layers of insulation between the Al RHE and the Au RTD at locations where the two intersected. Both the RHE and the RTD were cut into serpentine ribbons, which contributes to the stretchability and softness of the device. Specifically, the stretchability and softness of these serpentine ribbons can be maximized by fabricating their width to be as narrow as possible [37]. Due to the resolution of the Silhouette cutter, all ribbon widths were fixed to be 400 µm [42]. Although the resolution is far from photolithographic patterning technologies, the cost of time, materials and facilities is significantly reduced using the freeform cut-and-paste process because it does not require any chemicals, photomasks or cleanroom facilities. Moreover, while photolithographic process is limited to wafer scale, the patterning area of the cutter plotter can be as large as 30 cm. wide and a meter long.

Costs of previous epidermal electronic systems are dominated by fabrication processes such as spin coating, photolithography, wet and dry etching and transfer-printing. These methods require expensive clean-room fees and chemical purchases and they are also very time intensive. Using the “cut-and-paste” method allows for the fabrication of the presented device without any of those costs, making it significantly more cost effective than other similar epidermal electronics [38,42,44,45].

Finally, an ultrathin, ultrasoft double-sided tattoo adhesive was laid on top of the RHE and the RTD, providing a final layer of electrical insulation as well as increased adhesion between the skin and the patch. Snap button connectors were used to connect lead wires to both the RHE and the RTD (Supplementary Information, Figure S1).

**Figure 2:**
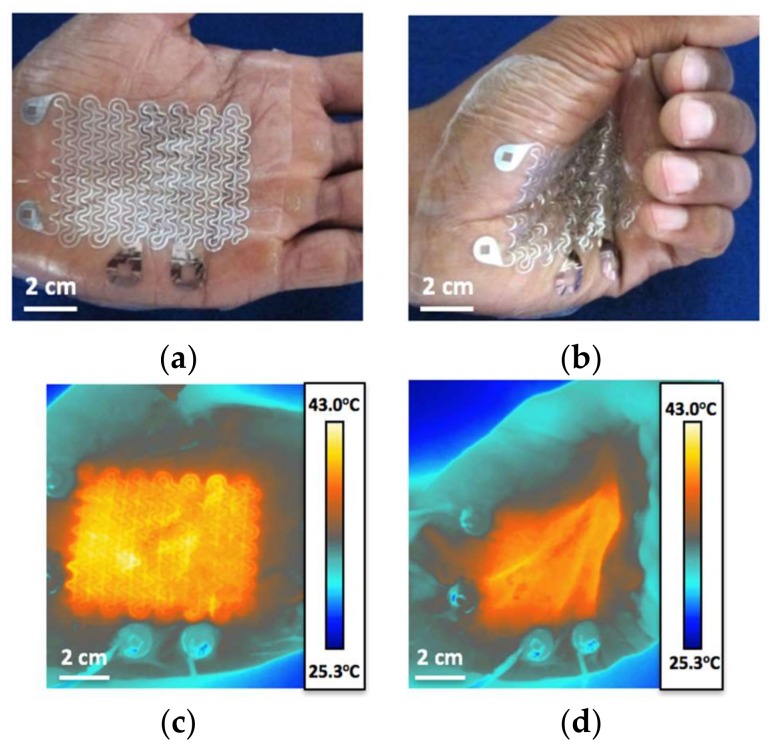
(**a**,**b**) Device conforms to hand and maintains its conformability during opening and closing; (**c**,**d**) Infrared (IR) images of the device powered with proportional-integral-derivative (PID) control as the hand is opened and closed. The PID controller automatically adjusts power output so the hand does not overheat when it closes.

The palm of a human subject’s hand was chosen for the location to test the device on. The palms of the hand are glabrous skin surfaces, and heating them along with the soles of the feet can efficiently warm the body during anesthesia [7,8]. Presenting that the heater works effectively on the palms of the hand therefore demonstrates that perioperative warming is a feasible application of this device. When attached to the skin, this device conformed to the skin and deformed alongside it without mechanical resistance, as evidenced by Figure 2a,b. When a DC voltage of 5.1 V was applied across the Al RHE, it supplied an even amount of heat over the palm around the target temperature of 40 °C. There was minimum change in temperature during severe skin deformation such as hand clenching, as demonstrated by Figure 2c,d, which were taken by an infrared (IR) FLIR T620 camera (FLIR, Wilsonville, OR, USA). The University of Texas at Austin IRB protocol number for the human subject experiments was 2010-03-0050.

**Figure 3:**
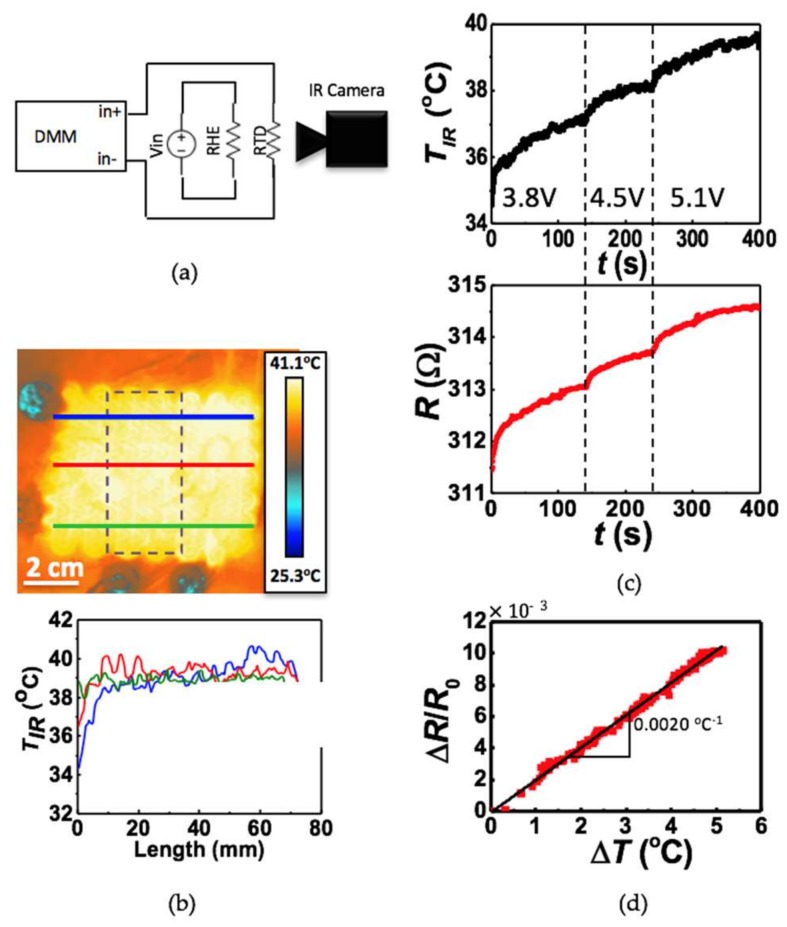
(**a**) Circuit diagram of set-up for calibration of RTD in situ. Heater is brought to different temperatures by adjusting Vin. Resistance and temperature are measured simultaneously using a digital multimeter (DMM) and IR camera, respectively; (**b**) Lateral heat distribution of heater. Blue, red and green lines on IR image mark the horizontal line across which temperature was measured for their respective red, blue and green plots. Temperature distribution is fairly uniform. Dotted purple line on IR image shows area that the IR camera calculated the average temperature for; (**c**) Average temperature of area marked by the dotted purple line in Figure 3b (top) and resistance of Au/Cr RTD as measured by DMM (bottom) each plotted across time as Vin was changed to 3.8 V, 4.5 V and finally 5.1 V; (**d**) The calibration curve for the RTD: ΔR/R_0_ of the RTD versus ΔT of the average temperature of the area around the RTD as marked by the dotted purple line in Figure 3b. The calibration constant, β, is marked and is equal to 0.000203.

To calibrate the RTD, it was placed on an insulated hot plate and its resistance was compared against the temperature readings of two custom made type T thermocouples. The RTD exhibited the expected linear relationship [15] between resistance and temperature with a temperature coefficient of resistance (TCR) of 0.0025 °C^−1^ (Supplementary Information, Figure S2). To obtain the TCR under service condition, calibration of the RTD was also conducted on skin together with the RHE, and an IR camera was used for temperature measurement. In this set-up, the heat comes from the RHE instead of the hotplate and the RTD is in intimate contact with the actual heat sink—the skin. Due to these differences, we hypothesized that the TCR would be different from that measured on the hotplate. A schematic of the calibration set-up is depicted in (Figure 3a). The RHE was linked to a DC voltage supply (Mastech Linear Power Supply HY1803D, Pittsburgh, PA, USA) while the RTD was connected to a digital multimeter (DMM, NI Elvis II). Resistance readings were logged using the DMM and LabVIEW 2014. The device was covered with a fine layer of Johnson’s Baby Powder to control its thermal radiation emissive properties [46]. The DC voltage supply was set to different voltages and the temperature and resistance of the RTD were measured simultaneously using the IR camera and the DMM, respectively. Temperature readings from the IR camera were logged using FLIR Tools+.

For safety purposes, the RHE-RTD calibration was first carried out on a glass slide, which has thermal properties similar to those of human skin [17]. The TCR was measured to be 0.0022 °C (Supplementary Information, Figure S3), which is slightly lower than that measured by the hotplate calibration. After ensuring the RHE behavior, a similar RHE-RTD calibration was performed on human skin. Figure 3b upper frame shows the IR image of the heater on human palm. It is evident that the temperature across the heater is fairly uniform over an area of 60 mm × 45 mm. The dotted black box indicates where the RTD resides. The IR temperature for the RTD calibration used in Figure 3c,d was obtained by averaging the temperature within this boxed area. It is clear in Figure 3b that the existence of the RTD does not affect the RHE or the temperature distribution. The three solid horizontal lines drawn across the heater mark the locations where the temperature is plotted as a function of distance along the lines in Figure 3b lower frame. Within the area covered by the RHE, temperature variation is between 38 °C and 40 °C. To continuously increase the temperature, the DC voltage was set to 3 increasing values: 3.8 V, 4.5 V and 5.1 V. Synchronously measured temperature and resistance versus time curves are provided in Figure 3c, which shows excellent alignment. Also visible in Figure 3c is the steady state average temperature of the RTD-area of the heater reaches when power is applied directly to the RHE. The average temperature to voltage ratio for the device was found to be 8.7 °C/V (Supplementary Information, Table S1). Plotting relative resistance change versus temperature change in Figure 3d, a linear fit with a TCR of 0.0020 °C^−1^ can be obtained. As expected, this is lower than the TCR found with the hotplate calibration (0.0025 °C^−1^) or the glass substrate calibration (0.0022 °C^−1^) due to the fact that the RTD is well conformed to human skin, beneath which blood flow can help mitigate the heat.

**Figure 4:**
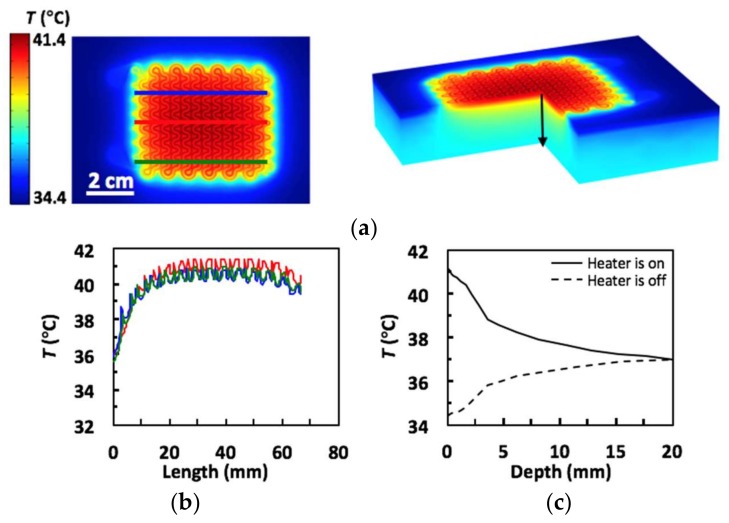
(**a**) COMSOL thermal simulation results (left: top view; right: 3D view); (**b**) Lateral heat distribution of heater. Blue, red and green lines on simulation image (left) mark the horizontal line across which temperature was collected for their respective red, blue and green plots; (**c**) Vertical heat distribution of skin from the black line on simulation image (right).

To verify the experimental findings, we ran a COMSOL simulation of the device heating human skin. The skin was modeled as a multilayer substrate made up of epidermis (0.1 mm thick), papillary dermis (0.7 mm thick), reticular dermis (0.8 mm thick), fat (2 mm thick) and muscle (16.4 mm thick), each with different thermophysical properties taken from literature [47]. No blood perfusion effects were included in the model. Ambient radiation from the RHE and convective cooling between the RHE and the environment were taken into consideration. With the environment temperature set at 15 °C and the core temperature set at 37 °C, the skin surface temperature stabilized at 34.4 °C when the heater was off. The effective electrical conductivity of the RHE was calibrated by setting the maximum temperature to be 41.4 °C when the applied voltage was 5.1 V. Using a Joule heating model for the RHE and a heat transfer model for the other components of the device and the skin, the modeled temperature distribution across the skin was found under transient and equilibrium states. Figure 4a,b displays the top and 3D cross-sectional views of the temperature distribution within the skin under equilibrium while the heater was on. Figure 4c plots temperature distributions along the three lines drawn on the left frame of Figure 4a where the blue, red and green curves correspond to the blue, red and green lines, respectively. The close agreement between Figure 3b and Figure 4c validates the COMSOL model and gives more credit to the simulated equilibrium temperature distribution in the skin along the depth direction (as indicated by the black arrow in Figure 4a right frame), as plotted in Figure 4c. When the heater is off (dashed curve), skin surface temperature is 34.4 °C. As the depth increases, the curve approaches the core temperature of 37 °C. When the heater is on (solid curve), skin surface is heated to 41.4 °C. The temperature gradually decays to 37 °C as we go deep into the skin. The slight kinks in the curves are due to the change of the thermophysical properties of the different layers of human skin.

**Figure 5:**
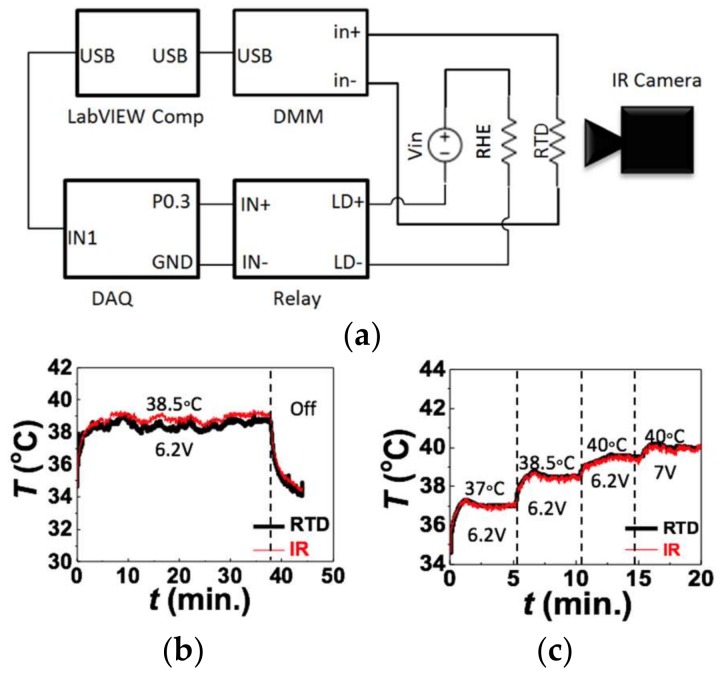
(**a**) Circuit diagram of set up for operating heater with PID control. DMM measures resistance of RTD and feeds it into a computer with LabVIEW, The LabVIEW program calculates the temperature of the RTD using the RTD’s starting temperature, starting resistance and calibration constant. It then uses a PID algorithm to calculate the optimal duty cycle for PWM of the heater given the heater’s current temperature and the set point temperature for the heater. The LabVIEW program then uses the data acquisition unit (DAQ) to switch the relay on and off with the determined duty cycle, thus controlling how much total power is fed to the heater; (**b**) Temperature of the heater versus time measured with both the RTD and the IR camera as the heater is turned on at a set point of 38.5 °C and then turned off. Heater is able to maintain set point temperature for an extended period of time; (**c**) Temperature of the heater versus time measured with both the RTD and the IR camera as the set point of the heater is changed while the voltage remains constant. At 40 °C, 6.2 V is not sufficient for the heater to reach the set point, so the voltage is increased to 7 V, at which point the heater is able to reach and maintain a temperature of 40 °C.

After calibrating the RTD and characterizing the RHE, we were able to establish a real time PID feedback control as illustrated by the diagram in Figure 5a. The purpose of this system was to demonstrate the functionality of the tattoo-like heater itself and was thus built with wires connecting the heater to a data acquisition (DAQ) unit and a PC. The system can be made to be wireless in the future by integrating it with a microcontroller unit (MCU), a Bluetooth low energy (BLE) chip and a rechargeable battery on a miniature printed circuit board (PCB). The DC power to the RHE was routed through an Omron DC-DC relay (G3CN) which was controlled by a computer using an output DAQ (NI USB-6009). The computer ran a LabVIEW program which controlled the temperature of the RHE using pulse width modulation (PWM). The RTD was connected to the DMM of an NI Elvis II, which measured the RTD’s resistance and sent the readings to the LabVIEW program in real time. The LabVIEW program converted the resistance readings into temperature using the following equation:

(1) $$T=T_{0}+\frac{\Delta R}{0.002R_{0}},$$

where the initial resistance $R_{0}$ was measured at the room temperature $T_{0}$ and the coefficient 0.0020 °C^−1^ was the TCR obtained from the calibration on human skin in Figure 3d. The PID program then used the real-time temperature feedback, along with a desired temperature set point, to determine how to control the relay and thereby the PWM of the RHE. This allowed the program to keep the heater at a set temperature or to adjust to a new temperature when demanded.

## 3. Results and Discussion

First, to test if the device could effectively maintain a target temperature the DC voltage supply was set to 6.2 V and the temperature was set to 38.5 °C. The device was able to maintain a constant temperature of 38.5 °C for over 30 min on human skin until the heater was completely turned off to finish the experiment as shown in Figure 5b. The heater reached the target temperature within 3 min and the error between the target temperature and the device’s temperature never reached more than a degree (Supplementary Information, Figure S4). The temperature readings of the RTD (black curve) was also verified by the IR camera results (red curve).

To test if the device could self-adjust when set temperature changes, we conducted an experiment with multiple set temperatures (37 °C, 38.5 °C, 40 °C) while the voltage supply was kept constant at 6.2 V (Figure 5c). For the first two temperatures (Stages I and II), the device was able to reach the set temperatures and to maintain them at a steady state. In switching between these temperatures, no changes were made except changing set point in the LabVIEW program. When the voltage was kept at 6.2 V and the target temperature was set to 40 °C, which is marked as Stage III, the actual skin surface temperature was not able to reach 40 °C. It could only reach up to 39 °C. This indicates insufficient power supply even when the duty cycle of the PWM reached 100%. Therefore, the maximum steady state temperature a heater can reach at 6.2 V with this set-up and under these circumstances is 39 °C, demonstrating a temperature to voltage ratio of 6.19 V/°C. This is lower than what was observed when the power was applied directly to the RHE and not routed through the relay. This indicates that some power may be lost as the electricity passes through the relay and the wires thereto. We therefore increased the voltage to 7 V and the skin surface was then successfully heated to 40 °C, as in Stage IV. Again, the temperature measured by the RTD (black) and the IR camera (red) are well matched. This experiment demonstrates that when given a sufficient voltage supply, the stretchable tattoo-like heater can automatically reach, maintain and change between desired temperatures without any manual adjustment of the voltage.

Due to the negligible stiffness of serpentine ribbons [48,49], the mechanical stiffness of our tattoo-like heater is dominated by the supporting Tegaderm tape, whose Young’s modulus was measured to be 7 MPa [42]. The effects of strain and skin deformation on our stretchable heater are discussed in Supplementary Information, Figures S5 and S6. Figure S5 indicates that our RTD can survive more than 70% tensile strain but its resistance is unfortunately slightly sensitive to strain. Due to such strain effects, Figure S6 indicates that the RTD temperature is not accurate when the hand closes but its measurement can recover when the hand restores its original configuration. With the PID control, the RHE may under heat the skin due to the falsely perceived increase of RTD temperature during hand closure, which will not cause any skin burn.

**Figure 6:**
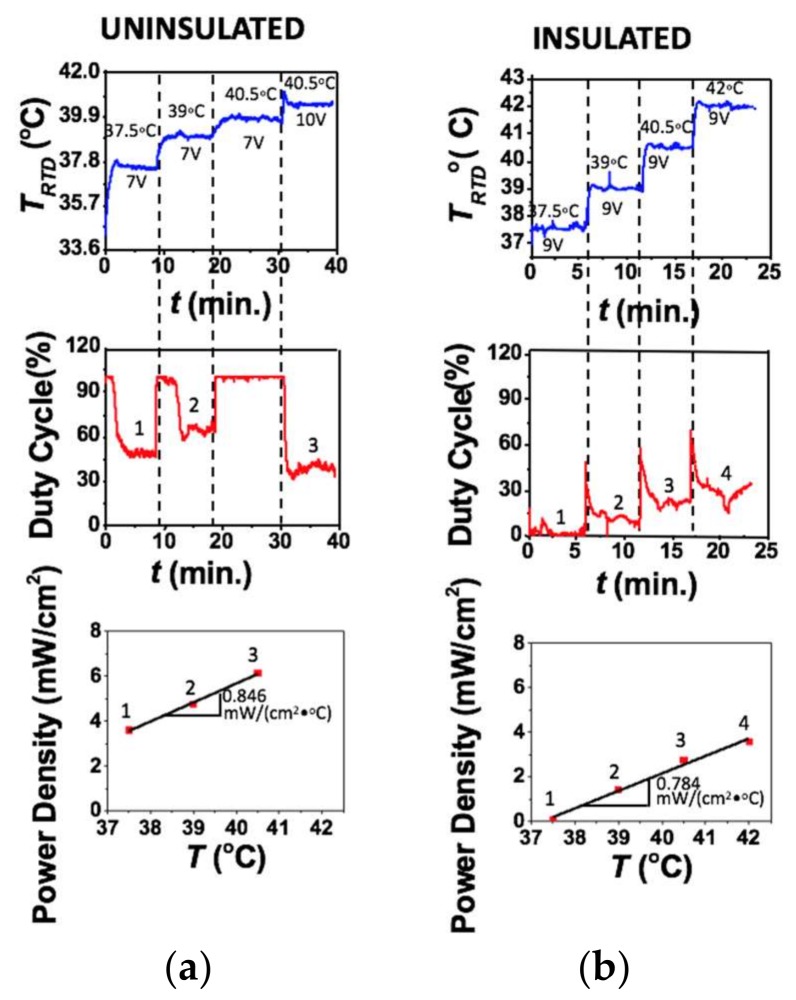
(**a**) Plot of heater temperature versus time as the set points and voltages are changed, followed by a plot of the corresponding duty cycle versus time. The average of the steady state duty cycles marked 1, 2 and 3 were used to calculate the power densities plotted below marked 1, 2 and 3, respectively, at different temperatures; (**b**) The same plots as figure A except the heater is insulated with a piece of foam.

To evaluate the power consumption of the tattoo-like heater, the duty cycles at different set temperatures were investigated. The device was placed on a human palm with PID control. The top frame in Figure 6a plots the actual skin temperature measured by the RTD versus time and the labels are again voltage supply and set temperature. Some small overshooting of the temperature occasionally happens at the points where voltage is changed but they are small and quickly rectified by the controller. The device is not expected to undergo step changes in voltages during real life application but these experiments show that the controller can react appropriately to those step changes should they occur. At each set temperature, the steady state duty cycle was recorded. The middle frame of Figure 6a shows the duty cycle versus time plot. The numbers mark the plateaus where the device was considered to have reached steady state. The duty cycle for each set temperature was calculated as the average of the duty cycle readings at these plateaus. The following equation was then used for power calculation:

(2) $$P=\frac{D}{100\%}\times \frac{V^{2}}{R},$$

where *D* is the duty cycle, *V* is the voltage supplied to the RHE and *R* is the resistance of the RHE.

If we define power density to be the power delivered to the skin per unit area of the RHE, power density can be calculated through:

(3) Power Density=P/A,

where *A* represents the total area of the heater, which is 38.7 cm^2^ for our RHE. Plotting power density versus the corresponding temperature as red markers in the bottom frame of Figure 6a, a linear relation can be fitted. The slope of this linear curve is defined as the specific power flow (SPF), which represents power density normalized to the applied thermal driving potential, that is, temperature difference. The SPF of our stretchable tattoo-like heater is estimated to be 0.846 mW/(cm^2^·°C), which means that to heat up a 1 cm^2^ area of this specific human palm by 1 °C would consume a power of 0.846 mW.

Considering convection and radiation between the heater and the ambient environment, it is inaccurate to assume that all the heat generated by the RHE completely goes into the skin. To obtain a more accurate estimation of the specific power flow into the skin, the entire experiment of Figure 6a was repeated in Figure 6b but with insulation over the heater. A 4 cm thick layer of foam, which is a well-known heat insulator, was taken from a delivery package and applied over the heater on the palm to minimize heat loss into the environment. With this heat insulating foam, the SPF was found to be 0.784 mW/(cm^2^·°C) as given in Figure 6b bottom frame, which is 7.33% lower compared with that of the exposed heater (0.846 mW/(cm^2^·°C)). This result indicates that about 7.33% of the heat generated by the RHE was lost to the environment when the RHE was exposed to air.

To compare our stretchable tattoo-like heater with other stretchable heaters in the literature, we summarized their materials, substrates, power densities and SPFs in Table 1. In cases where power supplied without using PWM, the value of *D* in Equation (2) was set to 100%. It is evident that the SPF of our tattoo-like heater is the lowest among the stretchable heaters directly applied on human skin, which is an active heat sink in comparison with air, polydimethylsiloxane (PDMS) and glass. Moreover, our device is one of the first to implement real time feedback control for stretchable heaters on human skin.

If adopted for commercialization, our device could very feasibly be converted into a wireless portable device with a battery-operated microcontroller as has been demonstrated for other joule heating devices reported in the Table 1 [5,30,31].

## 4. Conclusions

A low cost, low power consumption stretchable tattoo-like heater was fabricated to reliably warm the skin surface to a target temperature. The device combines a stretchable RHE and a stretchable RTD into a single unit through the cost and time effective “cut-and-paste” fabrication method. The device is thin (60 μm thick) and soft (7.4 MPa modulus) so that it can conform to the complex 3-D surface of palms and can deform and remain attached during hand flexure without perceivable mechanical resistance. The RHE is able to reach set temperatures with relatively even distribution, including during hand movement. The RTD can monitor the real-time temperature of the palm accurately, as verified by simultaneous IR measurements. Through PID temperature feedback control, the device is able to maintain set temperatures for extended periods of time and can automatically adjust to a different temperature if the set point is changed on the controller. The SPF of our device is comparable with reported stretchable skin-mounted heaters. Its simple circuit and program can easily be downscaled to a battery powered printed circuit board (PCB) and microcontroller, giving it potential for point-of-care applications.

## Figures and Tables

**Table 1:** Compiled information about different stretchable heaters.

Ref.	T Sensor on Site	Feedback Control	Target T (°C)	Resistive Heating Element	Substrate	V (V)	R (Ω)	Area (cm^2^)	P (W)	Power Density (mW/cm^2^)	SPF mW/(cm^2^·°C)
Our device	Yes	Yes	43	Al (9 μm)	Skin	10	17.2	38.7	2.38	61.44	0.78
[5]	No	No	43	Ag NW/SBS (18/82)	Skin	3.7	~2	91	~7	~80	0.9
[9]	Yes	Yes	40	Au (190 nm)	Pig Skin	12	95.9	<2.3	1.5	>652	>40 *
[15]	Yes	No	ΔT = 6	Au	Skin	--	--	0.64	0.01	20.31	3
[30]	Yes	Yes	250	Ag/Ethylene Glycol (50 wt %)	50 μm PI	4.5	0.75	--	27	650	3
[31]	No	No	40	CuZr nanotrough network	Skin	1.7	3.9	1.24	0.74	600	35
[39]	No	No	39	Ag NW /PDMS 132 mg m^−2^	Air	4	50	38.5	0.32	8.31	0.6
[39]	No	No	56	Ag NW /PDMS 396 mg m^−2^	Air	5	15	38.5	1.67	43.29	1
[41]	Yes	No	37	Cr/Au 7/70 nm	PDMS	4.4	550	9	0.04	3.91	0.8
[40]	Yes	Yes	31	PE Yarn with CuNW coating	Skin	1.4	1.66	30.16	1.18	39.12	5

* Assuming power for glass experiments was same as power for pig skin experiments.

## Supplementary Materials

The following are available online at http://www.mdpi.com/2072-666X/9/4/170/s1, Figure S1: Snap button connections, Figure S2: RTD calibrated on hotplate, Figure S3: RTD calibrated on glass with RHE, Figure S4: Error from set point during extended palm heating with device with PID control. Figure S5: Stretchability test, Figure S6: Effect of skin deformation, Table S1: Steady state temperatures for different voltages and temperature to voltage ratios, Video S1: Hand Clench Test.
