# Supplementary material for: Stretchable Tattoo-Like Heater with On-Site Temperature Feedback Control
Source: Micromachines (Basel). 2018 Apr 8;9(4):170. doi: 10.3390/mi9040170 (PMC6187738; doi:10.3390/mi9040170)
Supplement: Supplementary file 1 [file micromachines-09-00170-s001.zip › Supplementary Files/Supporting Information Resubmission.pdf]

Supporting Information

Stretchable Tattoo-Like Heater with On-Site Temperature Feedback Control

Andrew Stier[1], Eshan Halekote[1], Andrew Mark[1], Shutao Qiao[2], Shixuan Yang[2], Kenneth Diller[3], Nanshu Lu[1, 2, 3, 4*]

[1]Department of Electrical and Computer Engineering, [2]Center for Mechanics of Solids, Structures and Materials, Department of Aerospace Engineering and Engineering Mechanics, [3]Department of Biomedical Engineering, [4]Texas Materials Institute, the University of Texas at Austin, Austin TX 78712, USA.

Keywords: epidermal electronics, wearable heater, temperature sensor, feedback control

* Corresponding author: nanshulu@utexas.edu, 512-471-4208, 210 E. 24th St, Austin, TX 78712

Fabrication of Au/Cr/PET Laminate

13-µm PET (Goodfellow USA) was taped around a 3 in. long, 1 in. wide, and 1 mm thick glass slide then cleaned with acetone, IPA, and water, and then blown dry with compressed air. 10 nm of chromium and then 100 nm of Au were then thermally evaporated onto the PET. The tape was then removed from the PET, and the PET was unwrapped from the glass slides.

Fabrication of RHE and RTD

Schematics for the cut-and-paste process are shown in Figure 1A. To make the RHE, an Al/PET laminate (Neptco Inc.) was rolled onto thermal release tape (TRT, Semiconductor Equipment Corp., USA) with the PET side facing the TRT. The other side of the TRT was adhered on the cutting mat of a Silhouette Cameo electronic cutter plotter, which was then inserted into the machine. A 2D pattern designed in SolidWorks was imported into Silhouette Studios software and carved on the Al/PET laminate. The TRT was removed from the cutting mat and covered by a polymer liner removed from a Tegaderm tape (3M). The TRT was then placed on a hotplate for 5 minutes at a temperature of 120 ^{o}C to deactivate the adhesive. The liner helped keep the laminate from delaminating from the TRT as it lost adhesion. The TRT was then taken off the hotplate, the liner was removed, and tweezers were used to peel off all of the excess Al/PET from the TRT, leaving only the pattern as designed. The adhesive side of a Tegaderm patch was placed onto the pattern to peel it off the TRT. The Al/PET pattern was thus transferred to the Tegaderm with the Al side facing the Tegaderm adhesive and the PET side facing outward. The Au/Cr/PET laminate was then put through the same process with the RTD pattern cut into it, and transferred on top of the RHE with the PET side facing the Tegaderm adhesive and the Au side facing outward. The final device is shown in Figure 1B. Snap buttons sandwiching the lead wires to the device were

installed at the terminals of the RTD and RHE as illustrated by Supplementary Information Figure S1.

Connections to the Device

Secure connections to the device are essential for efficient heat delivery and accurate temperature measurements. Connections to the device are made using brass snap buttons (.130 series, Rome Fastener Sales Corporation). Each of the aluminum (Al) resistive heating element (RHE) and the gold (Au) resistance temperature detector (RTD) has two circular terminal pads with square openings for snap button installation, as shown in Fig. 1B. Holes aligned with the openings were also cut in the Tegaderm substrate. Figure S1 shows how lead wires were connected to the snap buttons. For the RHE (Figure S1A), the lead wire and the Al terminal were both sandwiched between the upper stud and the lower eyelet. An arbor press (Drake 1.5 Ton Arbor Press) was used to clamp the stud and the eyelet such that the lead wire formed a secure contact with the Al terminal and the snap button. For the RTD, the lead wire was soldered to the stud and the stud and the eyelet were pressed together, sandwiching the Au terminal. Alligator clips can then clip on the snap buttons to connect the RHE and RTD to the circuit.

Calibration of RTD on Hotplate

A hotplate (Fisher Scientific Isotemp Digital HotPlate Stirrer 11-200-49SH) was covered with a graphite sheet to facilitate lateral heat distribution. A 3M Micropore tape was applied on the surface of the graphite sheet for electrical insulation. The RTD was placed on the Micropore

tape along with two custom made type T thermocouples, as shown in Figure S2A. The hotplate was then set to multiple temperatures. At each temperature, the resistance of the RTD was measured by an ohmmeter (Fluke 87) and the temperature nearby was measured by the thermocouples. The relative change of resistance is plotted against the temperature in Figure S2B, which can be fitted by a linear curve. The slope of this linear curve is found to be 0.0025 $°C^{-1}$, which is the temperature coefficient of resistance (TCR) of our RTD.

Calibration of RTD on Glass with RHE Turned On

The patch integrated with RTD and RHE was attached to an 18 in. x 18 in. x 0.3 in. slap of glass, covered with a fine layer of Johnson's Baby Powder to increase its emissivity[1]. The RHE was powered with a DC voltage supply (Mastech Linear Power Supply HY1803D) of 7.3V. The temperature was measured with an IR camera (FLIR SC305). When the RHE reached the desired temperature of around 40°C, the image is given in Figure S3A. The temperature distribution across the black line in Figure S3A upper frame was plotted in Figure S3A lower frame. It is clear in both frames that there is a heat concentration towards the center of the RHE. Figure S3B shows that the resistance of the RTD and the maximum heater temperature was found to have a linear relationship with a TCR of 0.0022 $°C^{-1}$, which is slightly lower than the TCR of the RTD calibrated on a hotplate. This was expected because the temperature induced by the RHE was not as uniform as that on the surface of the hotplate. When the RHE and RTD were connected to the PID feedback control circuit shown in Figure 5A, with a voltage supply of 11V and a set temperature of 40 °C, Figure S3C demonstrates that the RHE was able to reach a temperature of 40°C and maintain the temperature consistently for over 40 minutes till the voltage supply was turned off.

Heating and Temperature Sensing on Human Skin

The subjects washed their hands and dried them thoroughly. Each subject's palm was rubbed with a paper towel to abrade dead skin cells from the surface. The hand was fixed to a custom slanted platform covered with foam taken from a delivery package for thermal insulation with the palm facing outward. The tattoo-like heater was then applied to the palm and was connected into the circuit shown in Figure 5A using alligator clips. The tattoo-like heater was then covered with a fine layer of baby powder to control the thermal radiation emissive properties. An IR camera was positioned on a tripod and aimed at the tattoo-like heater. The IR camera recording and the LabVIEW controlling and logging program were initiated simultaneously, and then the DC voltage supply for the heater was turned on. Target temperatures were set in LabView as desired for the experiment. The LabVIEW program acquired and logged electrical resistance signals from the RTD on the device and converted these readings to temperature using Equation (1) where T_0 is the hand's initial temperature, R_0 is the RTD's initial resistance, and 0.002 is the TCR of the RTD calibrated on the palm. The program also regulated the temperature of the heater by varying the power supply using PWM. Experiments were terminated by turning off the DC voltage supply.

Stretchability Tests

An RTD was pasted on a Tegaderm tape and the tape was clamped on a customized tensile tester. The resistance of the RTD was measured using a digital multimeter (Rigol DM3068) as the

RTD was stretched horizontally to a strain of 70% (Figure S5A) and relaxed back to a strain of 0% three times. The results are plotted in Figure S5B. It is clear that strain has an effect on the resistance of the RTD despite the serpentine design. The larger the tensile strain, the higher the resistance. It is also evident from the cyclic test that the resistance-strain curve is reversible. Since our PID program computes the temperature using measured resistance (Eq. (1)), stretching the skin would lead to increased resistance hence cause the program to overestimate the temperature of the device.

Effects of Skin Deformation

To examine the impact of skin deformation, an integrated RHE/RTD was fabricated and placed on a human palm. The device was first tested without PID control as shown in Figure 3A. The RHE was powered with a constant voltage of 3.8V, the RTD was connected to a digital multimeter (NI Elvis II), and the device was recorded with an IR camera (FLIR T620). The subject closed his hand as much as he could without blocking the IR camera and opened it again. The synchronously measured temperature by the RTD (black) and by the IR camera (red) is offered in Figure S6A. Before the hand closure, the RTD read the correct temperature which was in good agreement with the reading of the IR camera. When he closed his hand, the real (IR) temperature of the hand increased slightly due to the resistance increase in the RHE ribbon. However, the RTD read a much higher temperature increase compared with the IR temperature, due to strain-induced resistance change. Once the hand opens up again, the temperature of the hand returns to its original temperature. The RTD reading started to converge to the IR reading, but with some residual difference.

The device was then set up according to Figure 5A with PID control. The set temperature was 40 °C and the voltage supply was 10V. The hand was closed and opened twice in a way similar to that of the previous experiment. The temperature readings from the RTD (black) and the IR (red) are plotted in Figure S6B. Whenever the hand closes, the RTD overestimates the temperature of the device, and the controller responds by decreasing the duty cycle of the RHE and hence the actual temperature decreases. When the hand opens, the RTD reads the correct temperature, and the controller increases the power to the RHE to bring its temperature back up to 40°C. Although the temperature reading of the RTD always overestimates the actual temperature during hand closure, the PID control would only underheat the skin, which prevents the skin from getting burned. Once the hand restores its original configuration, the RTD temperature approaches the real temperature again.

References for Supporting Information

1. Methods of Increasing Emissivity in the Infrared Spectrum. Available at: http://www.optotherm.com/emiss-increasing.htm. (Accessed: 2nd May 2016)

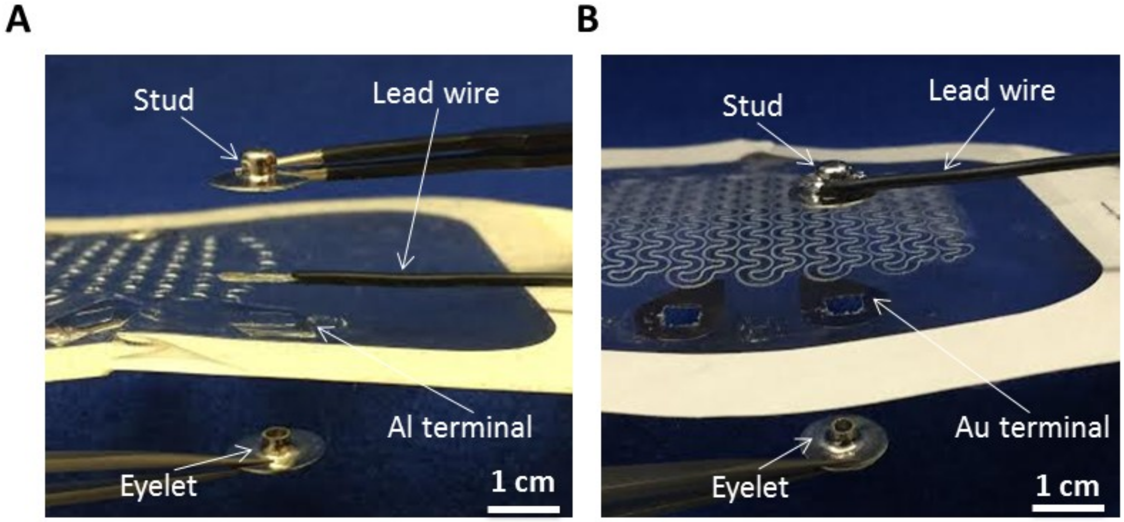

Figure S1: Snap button connections. (**A**) Snap button connection to the Al RHE with the lead wire sandwiched between the stud and the eyelet. (**B**) Snap button connection to the Au RTD with the lead wire soldered on the stud.

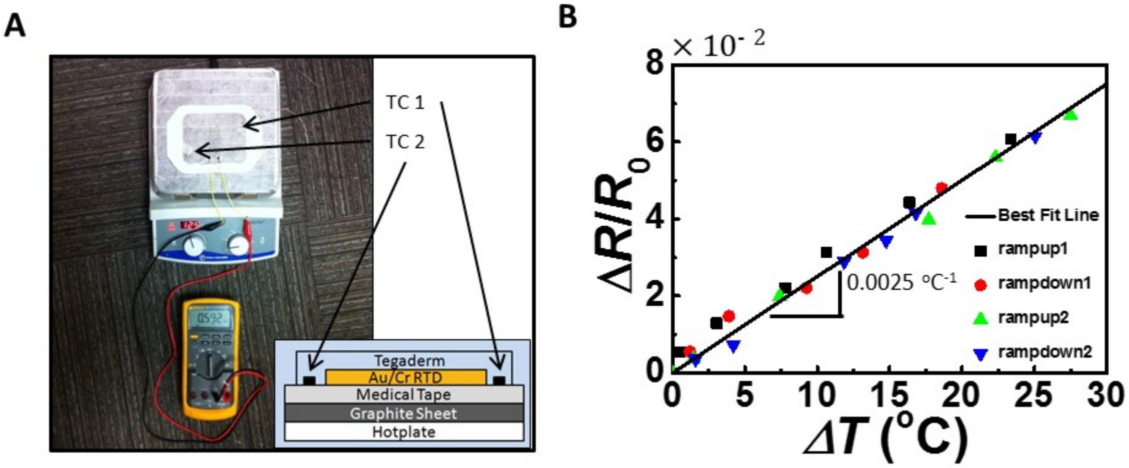

Figure S2: RTD calibrated on hotplate. (**A**) Set up for hotplate calibration of the RTD. Two thermocouples (TC1 and TC2) are placed near the RTD. The hotplate is set to various temperatures, and the resistance of the RTD and the temperatures of the thermocouples are recorded simultaneously. The inset shows a cross-sectional diagram of the set-up; (**B**) The calibration curve for the RTD: $\Delta R/R_0$ of the RTD vs. ΔT of the average temperature of the two thermocouples measured while ramping the temperature up and down twice. The slope of the curve, i.e., the temperature of resistance (TCR) of the RTD, is measured to be 0.0025 °C^{-1}.

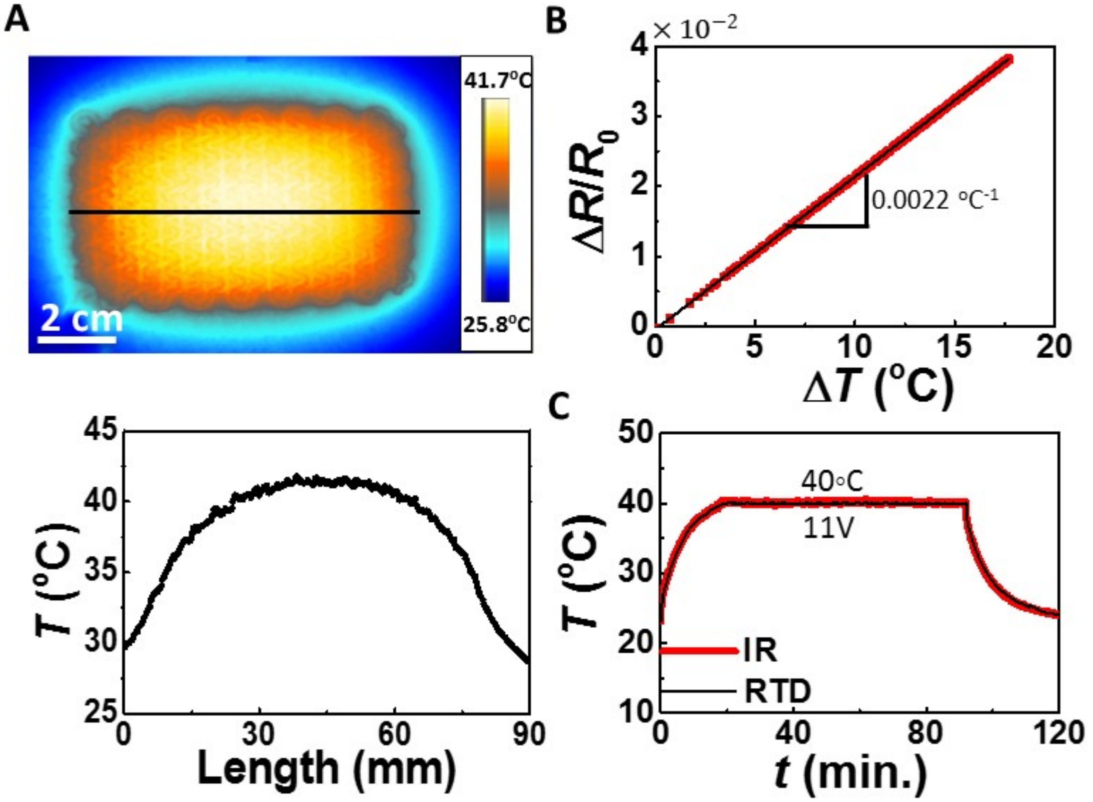

Figure S3: RTD calibrated on glass with RHE. (A) Top: IR image of the integrated RHE/RTD device on a 0.3 in. thick slab of glass. Bottom: lateral temperature distribution measured across the black line indicated in the IR image. (**B**) $\Delta R/R_0$ of the RTD vs. ΔT of the maximum temperature of the RHE. The TCR is found to be 0.0022 °C^{-1}. (**C**) When the device is connected to the circuit with PID control as illustrated by Figure 5A, the temperature of the RHE on glass was measured as a function of time using both the RTD (black) and the IR camera (red).

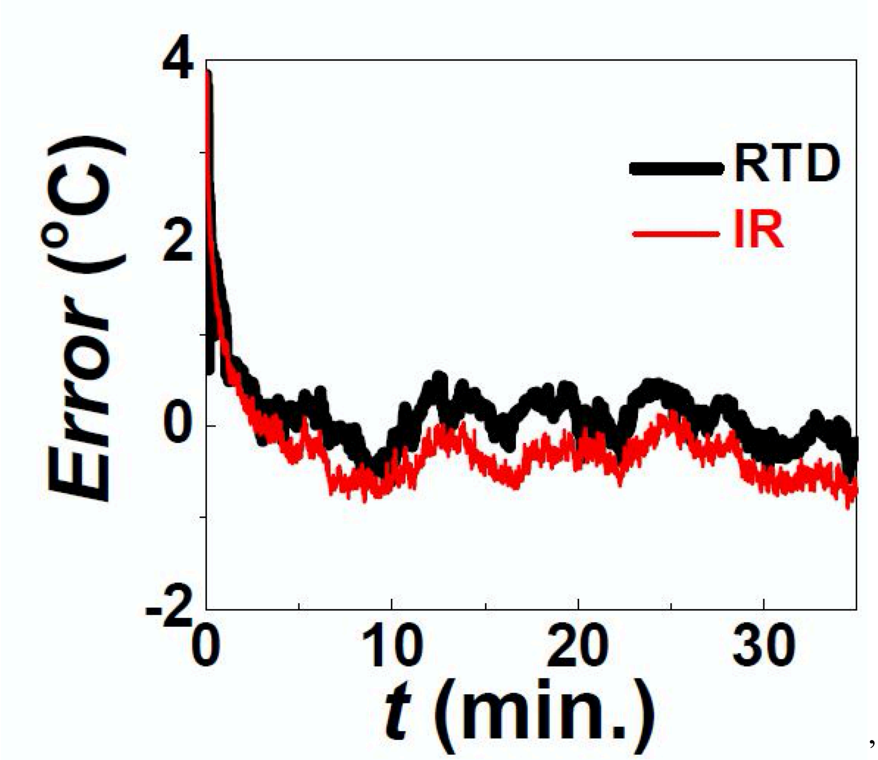

,

Figure S4: Error from setpoint during extended palm heating with device with PID control. The device reaches an error of 0 within 3 minutes, and the error is able to stay within a degree for the duration of the heating period.

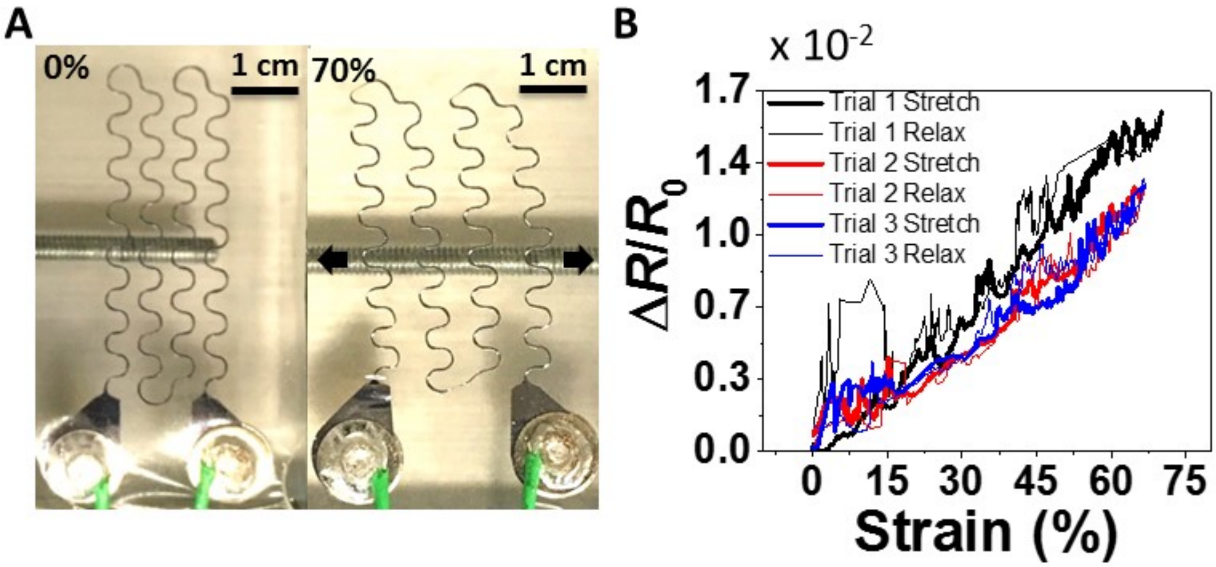

Figure S5: Stretchability test. (**A**) Pictures of the RTD before and after horizontal stretch. (**B**) Relative change of resistance as a function of applied strain during three stretch-unload cycles.

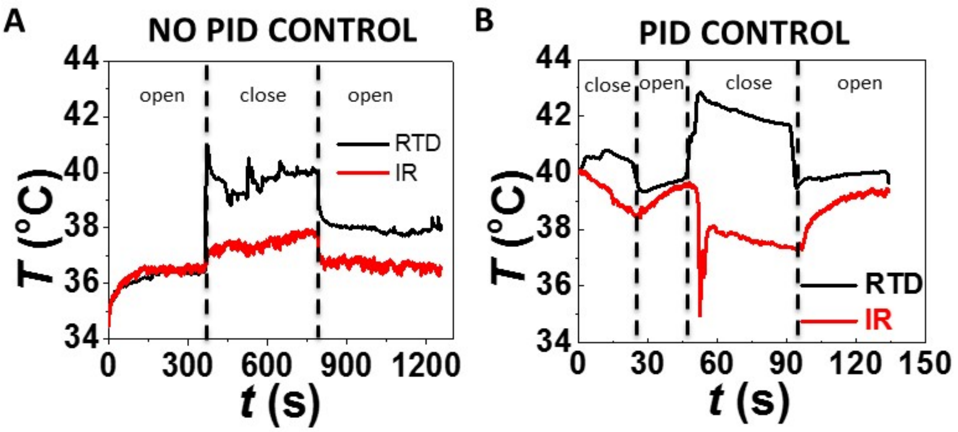

Figure S6: Effect of skin deformation. An integrated RHE/RTD device was applied on human palm. During hand closure, RTD resistance increased due to strain and hence RTD temperature was higher than IR temperature. (**A**) Under constant applied voltage but without PID control, the IR temperature (i.e. actual temperature) also increased during hand closure due to strain-induced increase of the RHE resistance. (**B**) With PID control and set temperature to be 40 °C, the IR temperature decreased during hand closure due to reduced power supply in response to the increased RTD temperature.

Table S1. Steady state temperatures for different voltages and temperature to voltage ratios

	**Voltage (V)**	**Steady State Temp (°C)**	**Temp/Voltage Ratio (°C/V)**
	3.8	37	9.7
	4.5	38	8.4
	5.1	39.5	7.8
**Average**			8.7
